# Editorial: Genetics and epigenetics of chronic kidney disease

**DOI:** 10.3389/fmed.2023.1078300

**Published:** 2023-02-13

**Authors:** Julia Hoefele, Jia Rao, Andrew J. Mallett

**Affiliations:** ^1^Institute of Human Genetics, Klinikum Rechts der Isar, School of Medicine, Technical University of Munich, Munich, Germany; ^2^Department of Nephrology, Children's Hospital of Fudan University, National Children's Medical Center, Shanghai, China; ^3^Shanghai Kidney Development and Pediatric Kidney Disease Research Center, Shanghai, China; ^4^Shanghai Key Laboratory of Birth Defect, Children's Hospital of Fudan University, Shanghai, China; ^5^Faculty of Medicine, University of Queensland, Brisbane, QLD, Australia; ^6^Institute for Molecular Bioscience, University of Queensland, Brisbane, QLD, Australia; ^7^College of Medicine and Dentistry, James Cook University, Townsville, QLD, Australia; ^8^Department of Renal Medicine, Townsville University Hospital, Townsville, QLD, Australia

**Keywords:** kidney genetics, epigenetics, nephrogenetics, genomics, chronic kidney disease

Diagnostics, therapy, and care for individuals affected by chronic kidney disease (CKD) are continuing to be better characterized, with increasing recognition of the resultant high health and economic burden worldwide due to CKD. Genetic contributors to CKD are also being better recognized and considered among both adults and children affected by CKD, with more than 500 different genes thus far identified to be associated with CKD. This Research Topic provides a concise overview of the current knowledge and outlook on future developments in diverse hereditary kidney diseases leading to CKD. The focus of this Research Topic is hereditary nephropathies including glomerulopathies (e.g., Alport syndrome and nephrotic syndrome), ciliopathies, tubulopathies, and congenital anomalies of the kidney and urinary tract (CAKUT). Here, we provide a broad overview of the articles included and reflect on the progress still to be made in this important area.

Alport syndrome is characterized by microscopic hematuria and proteinuria leading to end-stage kidney disease until 40 years of age. Male individuals are phenotypically more clearly affected because of X-linked inheritance in the majority of instances, although there is a more strongly emerging understanding of autosomal dominant forms. Female individuals with X-linked Alport syndrome usually show a milder phenotype compared to affected male individuals. CKD can also be observed in these individuals but is less frequent and/or occurs later in life. X-inactivation and other genetic modifiers have been postulated as being causative for the clinical variability. Günthner et al. showed that there is no correlation between the phenotype and X-inactivation, thus leading to the hypothesis that other genetic modifiers shape the phenotype in female individuals. Zhou et al. focused on the very rare occurrence of Alport syndrome with diffuse leiomyomatosis due to contiguous *COL4A5-COL4A6* gene deletions and showed that female individuals are mildly affected compared to male individuals. In addition, extrarenal manifestations, which are known to occur in individuals with Alport syndrome such as hearing impairment or ocular changes, have not been as frequently observed as in female individuals.

Nephrotic syndrome is one of the most frequent causes of glomerulopathy in children and is characterized by proteinuria, hypalbuminemia, and edema. In ~30% of children with steroid-resistant nephrotic syndrome (SRNS), a monogenic cause can be identified. Rong et al. retrospectively analyzed the genotypes and phenotypes of a Chinese cohort of pediatric individuals with disease-causing variants in *NPHS1*, one of the most mutated genes in individuals with SRNS. Next to the already known phenotypes, this study could identify three disease-causing variants in *NPHS1*, often observed in Chinese patients with congenital nephrotic syndrome implicating potential founder variants. Zhu et al. performed a genotype–phenotype correlation in a Chinese cohort of pediatric individuals and observed that almost 74% carry disease-causing variants in one of the following genes: *WT1, NPHS1, NPHS2*, and *ADCK4*.

Congenital anomalies of the kidney and urinary tract are an umbrella term for a broad spectrum of symptoms, ranging from mild forms like vesicoureteral reflux to severe forms like bilateral renal agenesis. Li Y. et al. identified that the candidate gene *Gen1* interacts with *Robo2* and that this was observed to be associated with CAKUT in a mouse model. Soraru et al. have then described a research study underway that will seek to identify potential monogenic etiologies in a cohort of patients experiencing kidney failure of uncertain cause. This is important given the concurrent reporting of several key case reports of rare instances of monogenic kidney diseases by Gambino et al., Li Q. et al., and Tian et al..

There have also been key contributions to understanding gene expression and its role in kidney disease phenotypes. Shi et al. reported on the methylomic landscape and CAKUT phenotypes, and this is complemented by Raghubar et al., who demonstrated the spatial transcriptomic landscape in healthy human and mouse kidney tissues.

Individuals with a CAKUT phenotype may occasionally have autosomal dominant polycystic kidney disease (ADPKD). This circumstance is already described as a phenocopy disorder. ADPKD is one of the most frequent causes of end-stage kidney disease in adults. A study by Xu et al. showed abnormalities of methylation in the tissue of individuals with ADPKD, implicating the finding of a biomarker as a prognostic factor for disease progression.

Conceptualizing the space more broadly, Shen et al. identified *IFT144* gene expression as a potential biomarker in lupus nephritis. Li M.-s. et al. reviewed lipoprotein glomerulopathy, which is most commonly associated with variants in *APOE*. Chaudhary et al. undertook a genome-wide association study and identified the interaction between *SMOC2* and *APOL1* in the development of progressive CKD.

The utilization and clinical utility of clinical genetic diagnoses for monogenic kidney disease is an area of substantial interest. Xiao et al. reported on the experiences of preimplantation genetic testing in their center for monogenic kidney disease during the past 20 years, which has the potential to inform real-world practice. The potential applicability of such approaches is further highlighted by the breadth of rare genetic forms of CKD, as indicated by individual case reports by Gambino et al. on a case of *TTC21B*-associated kidney and liver disease, Li Q. et al. on a multi-generational pedigree association of a *WT1* variant with IgA nephropathy, and Tian et al. reporting a *KCNJ1*-associated case of Bartter syndrome type II.

The variety of these contributions to the literature exemplifies the breadth and depth of research that is currently underway in understanding the genetics and epigenetics of kidney disease. There is likely to be a substantial number of additional reports on novel phenotypes to be reported in association with known genes, even as the number of novel genes to be reported is likely to continue to slow. This further highlights the importance of an integrative approach to future research efforts, especially in incorporating research for a better understanding of gene expression and regulation associated with kidney phenotypes as a parallel though related area to germline gene variation.

## Author contributions

JH, JR, and AM wrote the original draft and edited the manuscript. All authors reviewed and approved the final manuscript.

